# The Mbd4 DNA glycosylase protects mice from inflammation-driven colon cancer and tissue injury

**DOI:** 10.18632/oncotarget.8721

**Published:** 2016-04-13

**Authors:** Amy Marie Yu, Jennifer A. Calvo, Suresh Muthupalani, Leona D. Samson

**Affiliations:** ^1^ Biological Engineering Department, Massachusetts Institute of Technology, Cambridge, 02139, Massachusetts, USA; ^2^ Biology Department, Massachusetts Institute of Technology, Cambridge, 02139, Massachusetts, USA; ^3^ Department of Comparative Medicine, Massachusetts Institute of Technology, Cambridge, 02139, Massachusetts, USA; ^4^ Center for Environmental Health Sciences, Massachusetts Institute of Technology, Cambridge, 02139, Massachusetts, USA; ^5^ Koch Institute for Integrative Cancer Research, Massachusetts Institute of Technology, Cambridge, 02139, Massachusetts, USA

**Keywords:** Mbd4, colon cancer, inflammation, ulcerative colitis, AOM/DSS

## Abstract

Much of the global cancer burden is associated with longstanding inflammation accompanied by release of DNA-damaging reactive oxygen and nitrogen species. Here, we report that the Mbd4 DNA glycosylase is protective in the azoxymethane/dextran sodium sulfate (AOM/DSS) mouse model of inflammation-driven colon cancer. Mbd4 excises T and U from T:G and U:G mismatches caused by deamination of 5-methylcytosine and cytosine. Since the rate of deamination is higher in inflamed tissues, we investigated the role of Mbd4 in inflammation-driven tumorigenesis. In the AOM/DSS assay, *Mbd4^−/−^* mice displayed more severe clinical symptoms, decreased survival, and a greater tumor burden than wild-type (WT) controls. The increased tumor burden in *Mbd4^−/−^* mice did not arise from impairment of AOM-induced apoptosis in the intestinal crypt. Histopathological analysis indicated that the colonic epithelium of *Mbd4^−/−^* mice is more vulnerable than WT to DSS-induced tissue damage. We investigated the role of the *Mbd4^−/−^* immune system in AOM/DSS-mediated carcinogenesis by repeating the assay on WT and *Mbd4^−/−^* mice transplanted with WT bone marrow. *Mbd4^−/−^* mice with WT bone marrow behaved similarly to *Mbd4^−/−^* mice. Together, our results indicate that the colonic epithelium of *Mbd4^−/−^* mice is more vulnerable to DSS-induced injury, which exacerbates inflammation-driven tissue injury and cancer.

## INTRODUCTION

Ulcerative colitis (UC) is associated with substantially increased risk of colorectal cancer (CRC) [1], but not all UC patients will develop cancer [2]. It is known that particular mutations arise during the progression of cancer in UC patients [3], and recent genome wide association studies (GWAS) identified numerous polymorphisms that predispose a colitis patient to CRC [4]. Regardless of these advances, it remains difficult to predict whether an UC patient will develop colitis-associated CRC.

Inflammation is accompanied by the release of reactive oxygen and nitrogen species (RONS) that induce a wide variety of potentially carcinogenic chemical changes to DNA bases, including oxidation, alkylation and deamination [5–7]. The azoxymethane/dextran sodium sulfate (AOM/DSS) protocol is a model of inflammation-driven colon cancer that recapitulates many aspects of UC-associated carcinogenesis in human patients [8]. AOM is a colon-specific tumor initiator, and DSS elicits inflammation that promotes tumorigenesis. At low doses, AOM and DSS are only tumorigenic when used in combination [8]. AOM/DSS has been previously used to investigate the role of DNA damage response proteins in the development and progression of inflammation-driven colon cancer [9–16]. We recently reported that three DNA repair enzymes, namely, alkyladenine DNA glycosylase (Aag), Alkbh2, and Alkbh3, protect against tissue injury and tumorigenesis using this mouse model of inflammation-driven colon cancer [11, 16]. All three enzymes are known to repair RONS-induced DNA damage [16–21]. Here we ask if Mbd4, a DNA glycosylase involved in repair of deaminated cytosines, is also important for suppression of inflammation-mediated tumorigenesis.

Mbd4 (5-methylcytosine binding domain protein 4; also referred to as Med1) is a multidomain protein comprising a C-terminal monofunctional thymine-uracil DNA glycosylase and an N-terminal methyl-CpG binding domain [22]; reviewed in [23, 24]. The methyl-CpG binding domain of Mbd4 targets the enzyme to CpG-rich areas of the genome [25], where it initiates base excision repair (BER) at T:G or U:G mismatches produced by deamination of 5-methylcytosine (5MeC) or cytosine, respectively [22]. Hydrolytic deamination of 5MeC is a frequent occurrence in the genome [26], and its rate is increased by the RONS present in chronically inflamed tissues (reviewed in [27]). If left unrepaired, these mismatches result in C:G to T:A transitions at CpG sites. C:G to T:A transition mutations in tumor suppressor genes are a frequent feature of human cancers [28], suggesting that Mbd4-mediated repair may be important for suppressing carcinogenesis in chronically inflamed tissues. Supporting the idea that Mbd4 may participate in repair of RONS-induced DNA damage, Mbd4-depleted cells show heightened susceptibility to oxidative stress *in vitro* [29]. Mbd4 is upregulated at the protein level in hydrogen peroxide-exposed cells, and is subsequently recruited to a genomic area known to be sensitive to oxidative damage [29]. In humans, sequence alterations in MBD4 and altered *MBD4* expression are associated with elevated cancer risk. The MBD4 Glu346Lys polymorphism is associated with increased risk of colorectal cancer [30], esophageal squamous cell carcinoma [31], and lung adenocarcinoma [32]. *MBD4* has been observed to be epigenetically downregulated in colorectal and ovarian cancer [33], and low MBD4 expression significantly predicts reduced survival in colorectal cancer [34]. An MBD4 frameshift mutation resulting in a premature stop codon and truncated protein is frequently found in colon tumors [35, 36]; this truncated version of MBD4 impairs DNA repair in a dominant negative fashion [37]. Interestingly, polymorphisms in, or altered expression of MBD4 have also been observed in several autoimmune disorders, suggesting the possibility of a link between MBD4 status and inappropriate immune responses [38–42].

Previous studies in mice indicate that Mbd4 deficiency can interact with other factors to exacerbate gastrointestinal cancer. Although *Mbd4*-null mice do not have increased rates of spontaneous tumorigenesis, a null mutation in *Mbd4* on the *Apc^Min/+^* cancer prone background confers a further decrease in survival time and greater tumor multiplicity relative to the *Apc^Min/+^* single mutant [43, 44]. Mutations in the *Apc* gene in tumors from the *Mbd4^−/−^*/*ApcMin^/+^* mice show elevated C:G to T:A transitions at CpG dinucleotides, consistent with loss of Mbd4 repair activity [43, 44].

In addition to its role in base excision repair, Mbd4 has been implicated in several other processes that may influence the development of cancer. First, Mbd4 may promote apoptosis of cells with DNA damage. *Mbd4^−/−^* mouse embryonic fibroblasts are resistant to apoptosis induced by several chemotherapeutic agents [45]. In the small intestine *in vivo*, *Mbd4^−/−^* mice show reduced apoptosis after exposure to temozolomide, 5-fluorouracil, and cisplatin [46]. Mbd4 interacts with the Fas-activated death domain protein (FADD) protein, and may mediate apoptosis in response to a wide variety of stimuli, including Fas-ligand and detachment-induced apoptosis as well as apoptosis in response to DNA damage [47]. Mbd4 may mediate transcriptional repression [48], and possibly active demethylation of 5MeC [49–51], although this last role remains controversial [52, 53]; reviewed in [24]. Finally, Mbd4 interacts with and stabilizes the mismatch repair proteins Mlh1 [45, 54] and Msh2 [55], suggesting that Mbd4 may support mismatch repair integrity.

Here we show that Mbd4 suppresses morbidity and mortality in a mouse model of UC inflammation-associated colon cancer. *Mbd4^−/−^* mice have shorter survival times than wild-type (WT) mice with a greater tumor burden at time of death, and more severe clinical symptoms throughout the study. Histopathological examination of acutely inflamed WT and *Mbd4^−/−^* colons reveals greater inflammation and tissue damage in *Mbd4^−/−^* mice. Interestingly, we found no evidence that impaired apoptosis contributes to the increased tumor burden observed in AOM/DSS treated *Mbd4^−/−^* mice. Finally, we dissected the relative contribution of the *Mbd4* deficient colon and the *Mbd4* deficient immune system to the observed phenotype by transplanting WT bone marrow into *Mbd4^−/−^* mice. Transplantation of WT bone marrow into *Mbd4^−/−^* mice does not affect the decreased survival time or the intensified colonic injury induced by AOM/DSS; however, the tumor burden at death was similar between transplanted *Mbd4^−/−^* and WT mice.

## RESULTS

### *Mbd4^−/−^* mice have more severe clinical symptoms, reduced survival time, and increased tumor burden upon AOM/DSS treatment

**Figure 1:**
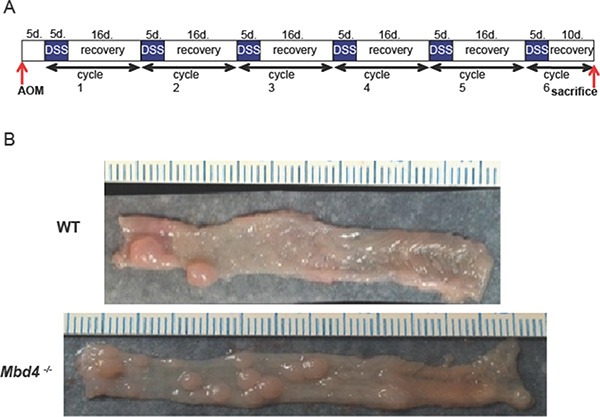
(**A**) The AOM/DSS protocol. Mice are injected with 12.5 mg/kg azoxymethane (AOM). Five days post injection, 1.5% w/v dextran sodium sulfate (DSS) is administered in the drinking water for 5 days. DSS water is then replaced with untreated tap water for 16 days to allow mice to recover. The combination of 5 days DSS and 16 days recovery comprises one “DSS/recovery cycle.” Mice are subjected to six DSS/recovery cycles to mimic the episodic flares and remissions associated with ulcerative colitis. Mice are sacrificed upon becoming moribund or on day 10 of the final recovery period. (**B**) Typical colons from AOM/DSS treated mice. Representative colons harvested from AOM/DSS treated WT (top) and *Mbd4^−/−^* (bottom) mice showing numerous polyps. Distal colon is to left. Scale is in millimeters.

To investigate the role of Mbd4 in inflammation-driven colon cancer, we compared the effects of AOM/DSS treatment on *Mbd4^−/−^* versus WT mice. In the AOM/DSS protocol (Figure 1A), tumors are initiated in the colon by a single injection (12.5 mg/kg) of the alkylating agent AOM. S_N_1 alkylating agents, such as AOM, result in the formation of several DNA base lesions including *O*^6^-methylguanine (*O*^6^MeG), which induces G to A transition mutations and apoptosis [56–58]. Tumor development is promoted by colonic inflammation that is induced via administration of the detergent DSS in the drinking water. DSS disrupts the colonic mucosa, allowing commensal bacteria to contact the underlying epithelial tissues, thus precipitating an inflammatory response [59, 60]. Periods of DSS administration (5 days) alternate with recovery periods (16 days) to mimic the flares and remissions associated with human UC. Importantly, neither AOM alone nor DSS alone, at the levels used in this study, is sufficient to induce tumors in WT mice [8]. Typical colon tumors induced by AOM/DSS in WT and *Mbd4^−/−^* mice are shown in Figure 1B; tumors were found almost exclusively in the distal half of the colon for both genotypes.

**Figure 2:**
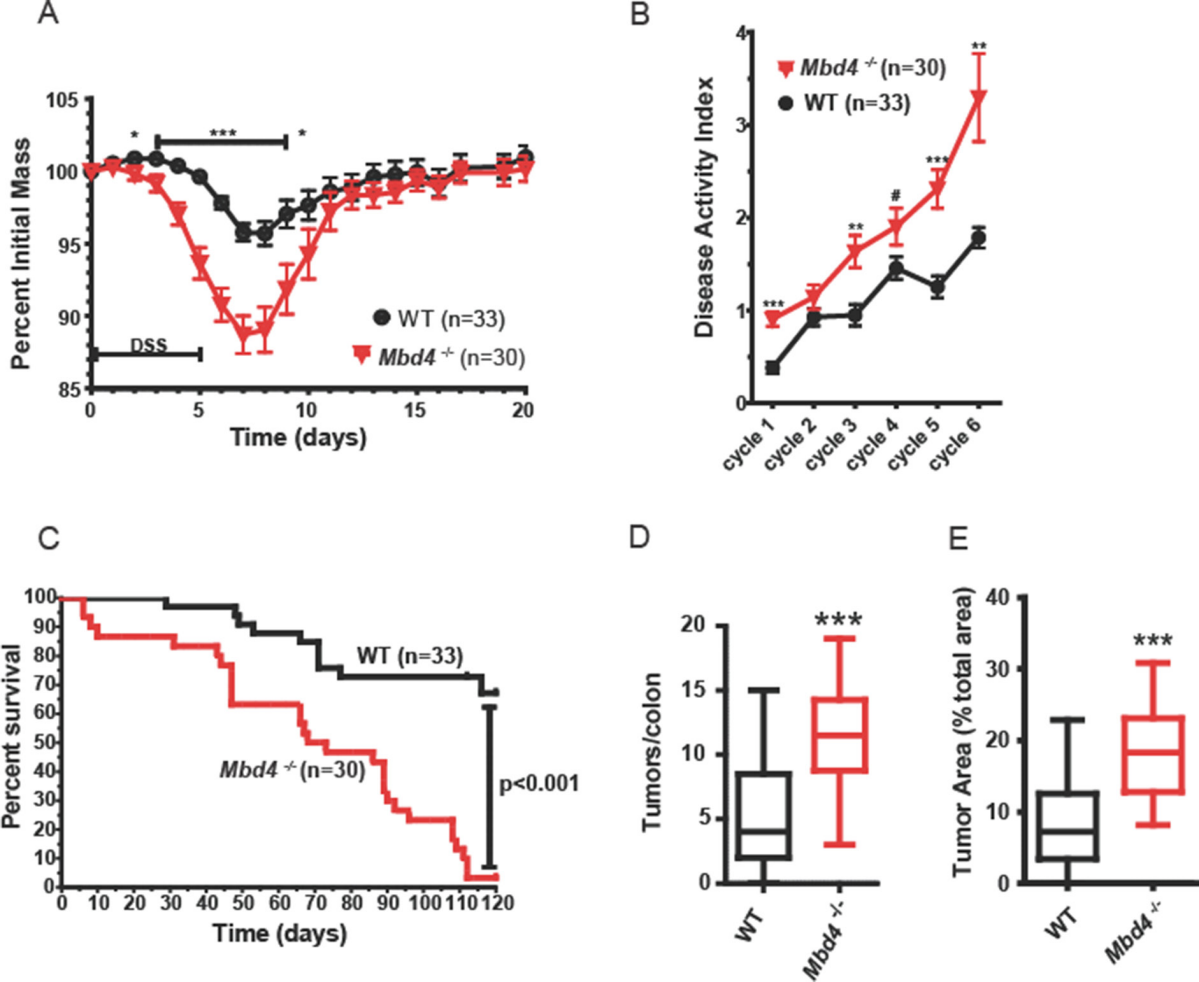
*Mbd4^−/−^* mice are more sensitive to AOM/DSS treatment (**A**) Body weight (BW) as a percentage of initial BW is shown during the initial AOM/DSS cycle for *Mbd4^−/−^* mice (red triangles, *n* = 30) and WT mice (black circles; *n* = 33). DSS was administered on days 0–5. (**B**) Mean disease activity index (DAI) score for *Mbd4^−/−^* mice (red triangles, *n* = 30) and WT mice (black circles; *n* = 33) during AOM/DSS treatment. DAI scoring criteria are detailed in Supplementary Table 1. (**C**) Kaplan-Meier survival plots of *Mbd4^−/−^* mice (red line) and WT mice (black line) during the AOM/DSS treatment. Note that four *Mbd4^−/−^* mice succumbed during the first round of DSS. (**D**) Tumor count per colon is shown for mice that survived the initial round of DSS administration for *Mbd4^−/−^* (*n* = 26) and WT (*n* = 30) mice. Box and whisker plots show minimum, maximum, and median. (**E**) Tumor area is illustrated for mice that survived the initial round of DSS administration, *Mbd4^−/−^* (*n* = 26) and WT (*n* = 30) mice. Tumor area is calculated as ratio of sum of all tumor areas to the total area of the colonic lumen using image analysis of photographs of whole mount colons.

When subjected to AOM/DSS treatment, *Mbd4^−/−^* mice lost substantially more weight than WT during the first DSS/recovery cycle, with significantly greater weight loss between day 2 and day 10 (Figure 2A). Disease activity index (DAI) scores, based on rectal bleeding, diarrhea and loss of overall condition (Supplementary Table 1), were also increased in the *Mbd4^−/−^* mice throughout the experiment (Figure 2B). Consistent with decreased body weight and increased DAI scores, survival was highly significantly reduced in AOM/DSS treated *Mbd4^−/−^* mice relative to WT (Figure 2C). In fact, four *Mbd4^−/−^* mice succumbed during the first DSS/recovery cycle and none of the *Mbd4^−/−^* mice survived by the end of the experiment. Tumor burden at time of death was significantly greater in *Mbd4^−/−^* versus the WT mice, measured both as number of polyps per colon (Figure 2D) and by the ratio of tumor to non-tumor area in the colonic lumen (Figure 2E). Histopathological analysis comparing colons from these AOM/DSS treated WT and *Mbd4^−/−^* mice showed no differences in epithelial defects, neoplasia area, crypt atrophy, or hyperplasia. Inflammation and stage of neoplasm were very modestly but significantly decreased in *Mbd4^−/−^* relative to WT colons (Supplementary Figure 1). Taken together, the more severe clinical symptoms, reduced survival, and greater tumor burden in AOM/DSS treated *Mbd4^−/−^* versus WT mice suggest that the Mbd4 protein plays a role in suppressing inflammation-driven colon carcinogenesis.

### Response to AOM is not altered in *Mbd4^−/−^* mice

**Figure 3:**
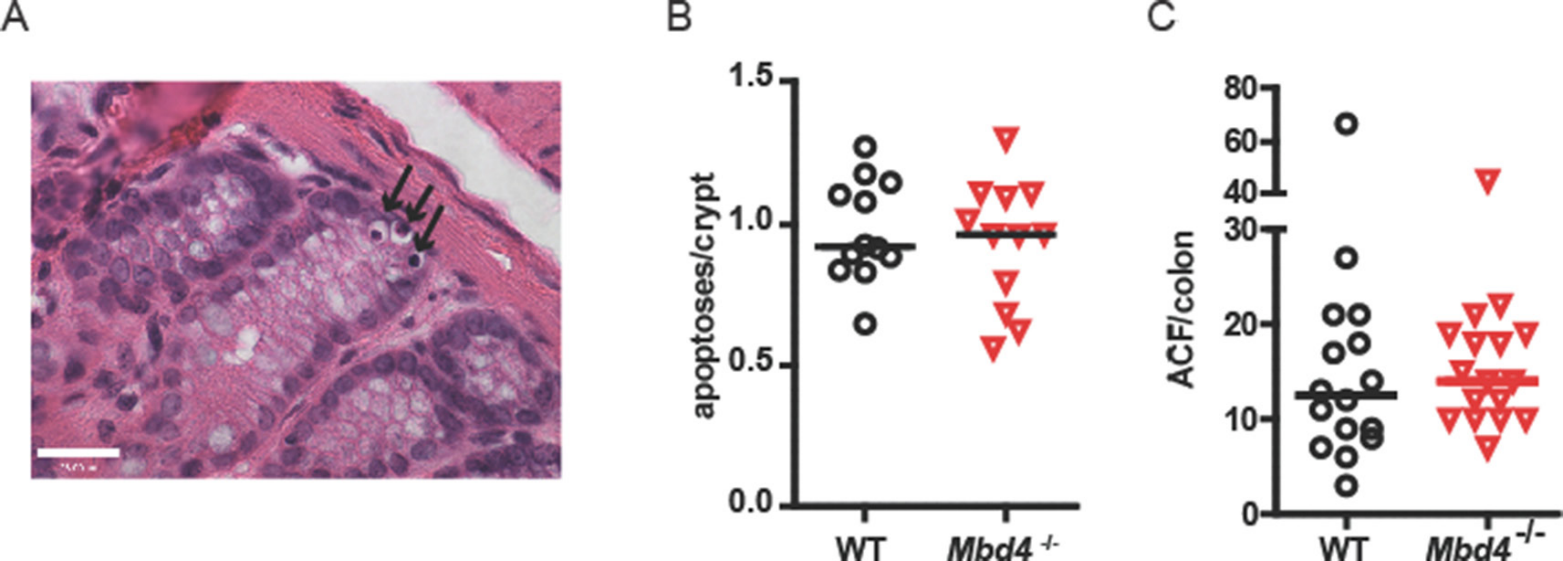
WT and *Mbd4^−/−^* mice exhibit similar extent of apoptosis in colon following AOM treatment (**A**) Representative apoptotic colonic crypt cells in an AOM-treated mouse is shown. Three apoptotic cells (black arrows) are visible as shrunken dark (pyknotic) nuclei at the bottom of a crypt in this H&E stained section. Magnification is 60×. Scale bar shows 23.00 μm. (**B**) Median number of apoptotic cells per crypt is shown for WT (*n* = 12) and *Mbd4^−/−^* (*n* = 12) mice 48 hours after injection of 12.5 mg/kg AOM. Median values are not significantly different (*p* > 0.83). (**C**) Aberrant crypt foci (ACF) in colons from WT (*n* = 16) and *Mbd4^−/−^* mice (*n* = 17) 18 weeks after a single injection of 12.5 mg/kg AOM. Median values are not significantly different (*p* > 0.40). Lines in diagram indicate median values.

To investigate possible mechanisms for increased tumorigenesis observed in AOM/DSS treated *Mbd4^−/−^* mice, we examined the effect of AOM alone on WT and *Mbd4^−/−^* colonic epithelium. Increased tumorigenesis may result from the failure of damaged *Mbd4^−/−^* colonic epithelial cells to undergo apoptosis, as previously reported for *Mbd4^−/−^* MEFs [45, 47] and small intestinal epithelial cells [46]. In WT mice, a single administration of AOM results in a wave of apoptosis in the intestinal epithelium, peaking 48 hours after AOM administration [13]. We quantitated colonic apoptosis in AOM-treated WT and *Mbd4^−/−^* mice 48 hours after AOM injection (12.5 mg/kg); this time point allowed two cell cycles post-treatment, necessary for AOM-induced apoptosis [61, 62]. Apoptotic cells were identified as pyknotic nuclei in H&E stained sections (Figure 3A). There was no difference in AOM-induced apoptosis between WT and *Mbd4^−/−^* colons (Figure 3B), suggesting that the increased tumor burden observed in AOM/DSS treated *Mbd4^−/−^* mice is not due to differential AOM sensitivity of the colonic epithelium.

As another measure of AOM sensitivity, we monitored the ability of a single dose of AOM (12.5 mg/kg) to produce precancerous aberrant crypt foci (ACF) in WT and *Mbd4^−/−^* mice. ACF appear as small areas of raised, thickened epithelium that stain intensely with methylene blue, often having abnormal slit-shaped crypt openings [63]. Eighteen weeks post injection, ACF were counted in methylene blue-stained whole mounts under a stereomicroscope. Consistent with Mbd4 having no effect on AOM-induced apoptosis, we observed no difference in the number of AOM-induced ACF in WT versus *Mbd4^−/−^* mice (Figure 3C). Taken together, these results suggest that the greater tumor burden in AOM/DSS treated *Mbd4^−/−^* mice is not due to increased tumor initiation by AOM.

### DSS treatment produces more severe tissue damage in *Mbd4^−/−^* versus WT mice

The severe clinical symptoms exhibited by *Mbd4^−/−^* mice during the first DSS/recovery cycle, before tumors could develop, led us to further investigate the acute effects of DSS on the *Mbd4^−/−^* colon. The severity of DSS injury was examined histopathologically over the course of a single DSS/recovery cycle in WT and *Mbd4^−/−^* mice. Colons were harvested on day 2 of DSS administration (before appearance of clinical symptoms in either genotype), day 4 of DSS administration (coincident with appearance of symptoms in *Mbd4^−/−^* mice), day 1 after DSS withdrawal (coincident with maximum symptom severity) and day 5 after DSS withdrawal (coincident with abatement of clinical symptoms). H&E sections of colons were scored for inflammation, epithelial damage, crypt atrophy, epithelial hyperplasia, and neoplasia severity and extent.

**Figure 4:**
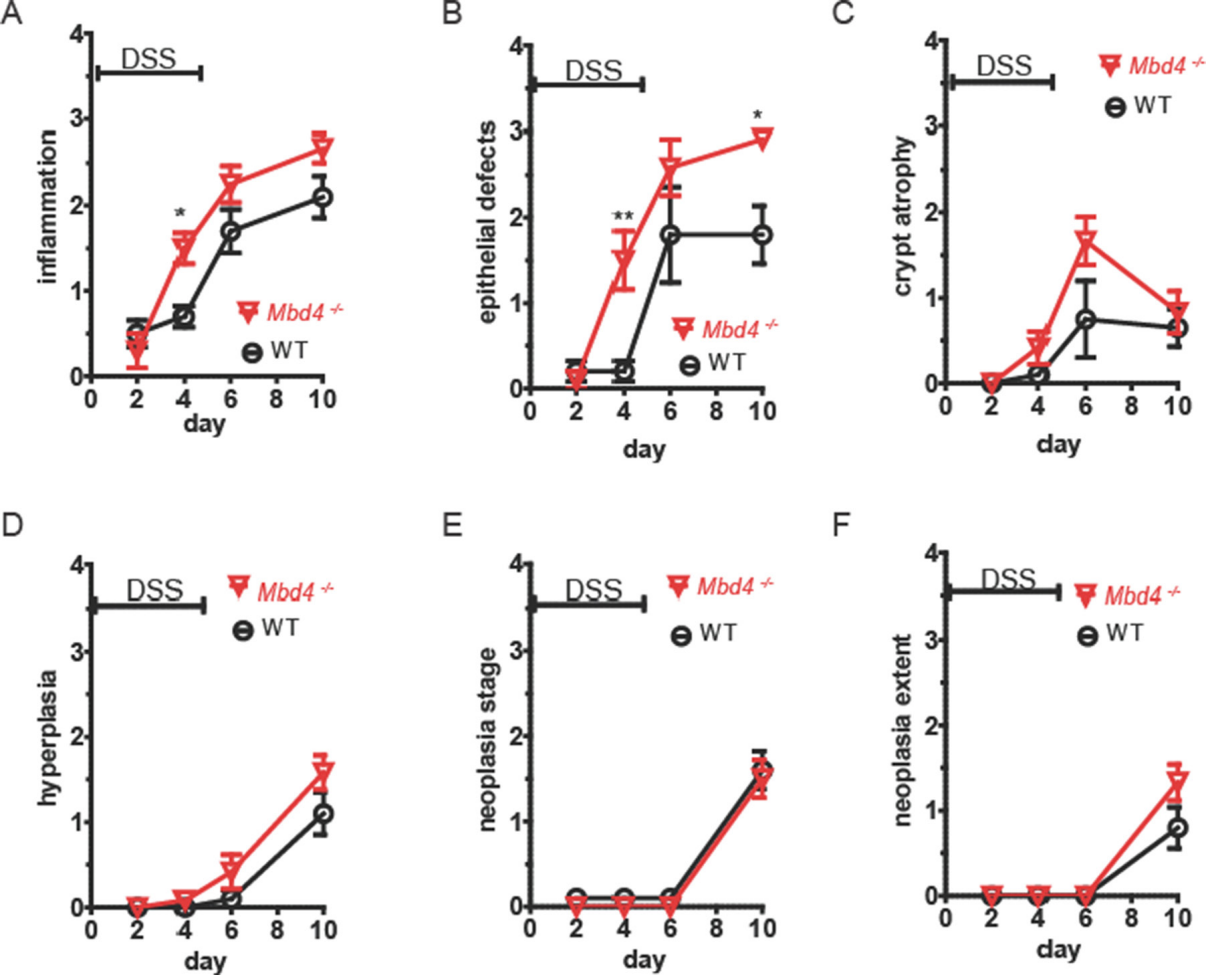
*Mbd4^−/−^* mice experience greater tissue damage from DSS treatment WT (black circles) and *Mbd4^−/−^* (red triangles) were treated with 1.5% DSS in drinking water on days 0–5, *n* = 5 or 6 all time points. Colons were harvested on the days indicated above and scored for pathology in H&E stained sections by a veterinary pathologist. Scores ranged from 0 (no pathology) to 4 (severe pathology) for the following criteria: (**A**) inflammation, (**B**) epithelial defects, (**C**) crypt atrophy, (**D**) hyperplasia, (**E**) neoplasia stage, and (**F**) extent of neoplasia.

Consistent with the greater severity of clinical symptoms in AOM/DSS treated *Mbd4^−/−^* mice, DSS-treated *Mbd4^−/−^* colons displayed an accelerated appearance of tissue damage and overall trends towards increased pathology relative to WT. Both inflammation and epithelial damage (Figure 4A and 4B) were significantly increased in *Mbd4^−/−^* colons relative to WT on day 4 (*p* < 0.02 and *p* < 0.007, respectively). Epithelial damage remained significantly increased in *Mbd4^−/−^* mice relative to WT five days after DSS withdrawal (*p* < 0.045). On average, colons from DSS-treated WT mice exhibited isolated foci of moderate epithelial attenuation, consistent with their relatively mild clinical symptoms. In contrast, *Mbd4^−/−^* mice showed extensive areas of mucosal necrosis. *Mbd4^−/−^* mice also showed trends towards increased scores for crypt atrophy (Figure 4C), hyperplasia (Figure 4D) and extent of neoplasia (Figure 4F). No difference was observed in neoplasia stage during the acute DSS exposure (Figure 4E). We infer that *Mbd4^−/−^* mice are more susceptible than WT to DSS-induced colonic tissue injury, and that this heightened susceptibility contributes to the increased clinical symptoms and reduced survival seen in AOM/DSS treated mice.

### Adoptive transfer of WT hematopoietic cells into *Mbd4^−/−^* mice

DSS-mediated tissue injury is a complex process involving an interaction of the immune system with colonic epithelial tissue [64]. The greater tissue damage seen in *Mbd4^−/−^* mice consequent to DSS treatment could be due to either a greater susceptibility of colonic epithelium to RONS-induced damage, a more robust and damaging innate immune response in *Mbd4^−/−^* mice, or both. It has previously been shown that mice lacking the DNA repair protein Atm manifest greater systemic inflammation in response to DSS exposure [14]. We therefore investigated whether transplantation of a wild-type immune system into the otherwise *Mbd4^−/−^* mice would affect the extent of AOM/DSS induced pathology.

**Figure 5:**
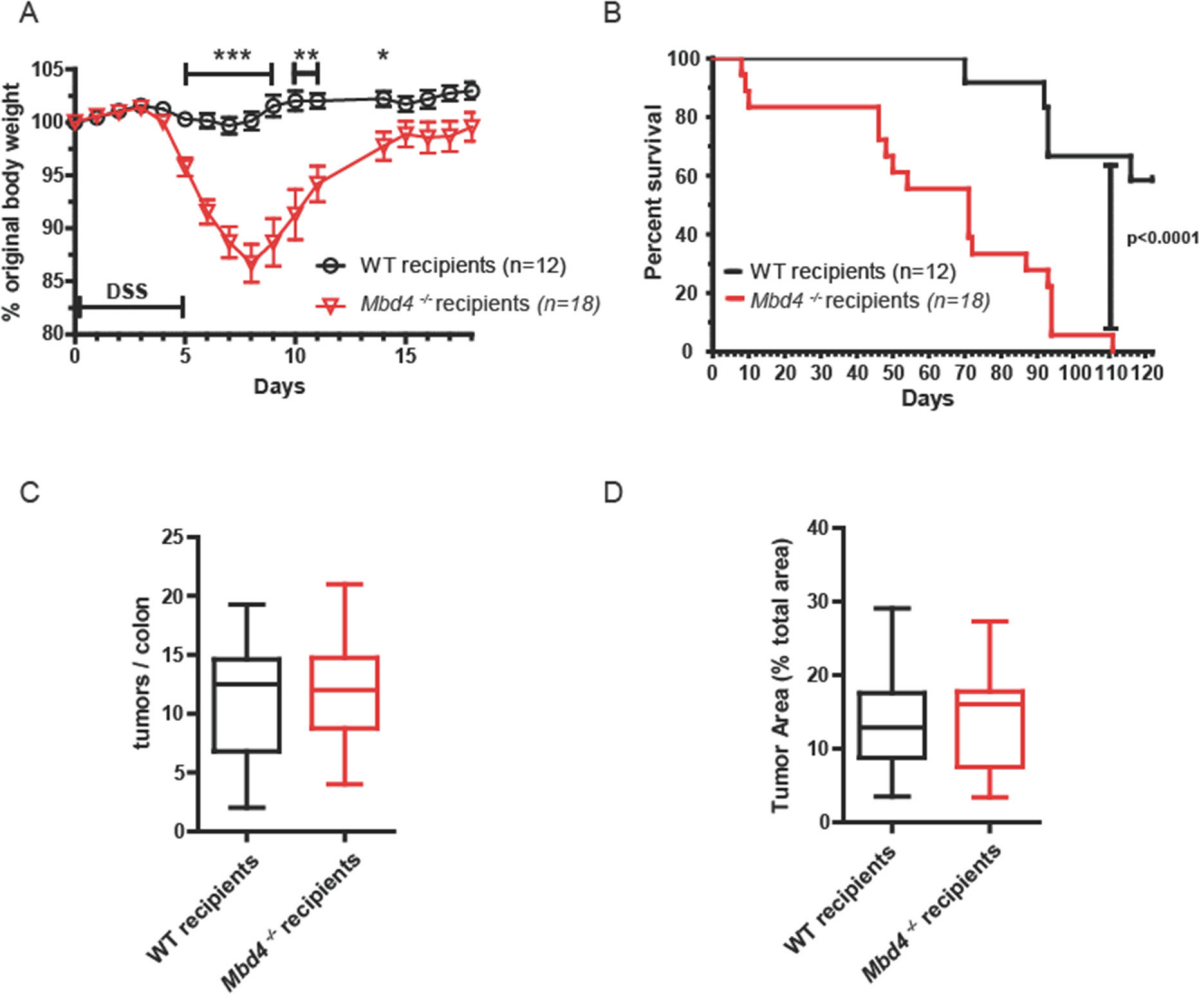
Adoptive transfer of WT hematopoietic cells into *Mbd4^−/−^* recipients protected against colon tumor formation, but did not affect overall survival (**A**) BW, shown as percentage of initial BW, is shown for WT recipients (*n* = 12) and *Mbd4^−/−^* recipients (*n* = 18) during AOM/DSS treatment. (**B**) Kaplan-Meier survival plot of WT recipients (*n* = 12, black line) and *Mbd4^−/−^* recipients (*n* = 18, red line) during AOM/DSS treatment. (**C**) Tumor multiplicity at time of death is shown for transplanted WT (*n* = 12) and transplanted *Mbd4^−/−^* (*n* = 15) mice. Difference in tumor multiplicity between genotypes is not significant (*p* > 0.8). (**D**) Tumor area is illustrated for transplanted WT (*n* = 12) and transplanted *Mbd4^−/−^* (*n* = 15) mice. Difference in tumor area between genotypes is not significant (*p* > 0.9). Tumor area is calculated as ratio of sum of all tumor areas to the total area of the colonic lumen using image analysis of photographs of whole mount colons.

We replaced the hematopoietic tissues of *Mbd4^−/−^* mice by adoptive bone marrow transfer from *B6.SJL-Ptprc^a^ Pep3^b^/Boy^J^* mice that are genetically identical to WT C57BL/6 mice with the exception of the allele present at the CD45 locus that allows differentiation between host and donor cells by immunostaining and flow cytometry. WT C57BL/6 mice that underwent the same reconstitution procedure (transplanted WT) were used as controls. Neither WT nor *Mbd4^−/−^* mice experienced substantial mortality from the adoptive transfer process; survival of the adoptive transfer procedure ranged from 70–100% for both genotypes, in accordance with previously reported survival rates [65]. Transplantation success was confirmed by peripheral blood analysis four weeks after the procedure. In both genotypes, 75–95% of peripheral blood mononuclear cells (PBMCs) and on average ∼60% of T cells were donor derived (Supplementary Figure 2). To assess long-term replacement of hematopoietic tissue, we followed small cohorts of transplanted WT and transplanted *Mbd4^−/−^* mice for approximately 6 months. By 19 weeks, the mean percentage of donor total PBMCs and T cells had increased to > 90% in all mice, and this level remained stable until the mice were sacrificed 27 weeks post transplantation (Supplementary Figure 2). Since the duration of a full-length AOM/DSS protocol is 18 weeks, we were confident that the stability of the graft over time was more than adequate. Colons of a subset of transplanted mice were examined 27 weeks post transplantation, and no tumors or abnormalities were visible in the colons of either genotype. Adoptive transfer of WT bone marrow in *Mbd4^−/−^* mice did not affect the clinical symptoms or survival in the AOM/DSS assay. Similar to non-transplanted *Mbd4^−/−^* mice, AOM/DSS treated transplanted *Mbd4^−/−^* mice lost a significantly greater percentage of initial body weight compared to their transplanted WT counterparts during the first DSS/recovery cycle (Figure 5A), and transplanted *Mbd4^−/−^* mice also showed greater lethality than transplanted WT controls in the AOM/DSS assay (Figure 5B). As with *Mbd4^−/−^* mice, transplanted *Mbd4^−/−^* mice began dying during the first DSS cycle, and none of the transplanted *Mbd4^−/−^* mice survived to the end of the experiment. The difference in survival was again highly statistically significant (*p* < 0.0001). In this experiment, there was no difference in tumor burden at time of death between transplanted WT and transplanted *Mbd4^−/−^* mice, as assessed by both tumor multiplicity (Figure 5C) and tumor area (Figure 5D). However, this lack of difference can be attributed to increased tumor burden in the transplanted WT mice relative to the tumor burden in the non-transplanted WT mice (compare Figure 2D, 2E and Figure 5C, 5D).

## DISCUSSION

Despite the extensive associations between the MBD4 DNA glycosylase and human cancer, the specific context in which MBD4 is important for suppressing tumors has remained unclear. *Mbd4^−/−^* mice were previously shown to have no change in the rate of spontaneous tumorigenesis versus WT mice [43], although loss of Mbd4 did accelerate tumorigenesis in the *Apc^Min/+^* cancer-prone model [43, 44]. Here, we show that *Mbd4^−/−^* mice exhibit greater morbidity and mortality in response to colonic inflammation and that this is accompanied by increased tumor burden versus WT controls.

*Mbd4^−/−^* mice are substantially more susceptible than WT mice to DSS-induced injury of the colonic epithelium. It is tempting to compare this with another recent result from our lab, which showed that triple knockout mice lacking the Aag DNA repair glycosylase and the DNA repair dioxygenases Alkbh2 and Alkbh3 are unable to survive even a single round of DSS treatment [16]. However, despite the fact that in both cases the DNA repair deficiency results in heightened susceptibility to DSS, there are notable differences between the phenotypes of AOM/DSS treated *Mbd4^−/−^* mice and *Aag^−/−^Alkbh2^−/−^Alkbh3^−/−^* mice. The degree of tissue damage in DSS-treated *Aag^−/−^Alkbh2^−/−^Alkbh3^−/−^* mice is not significantly greater than that in WT or single mutant mice; however, the *Aag^−/−^Alkbh2^−/−^Alkbh3^−/−^* mice are unable to regenerate new colonic epithelium after DSS withdrawal and eventually succumb following DSS treatment. In contrast, the *Mbd4^−/−^* mice sustain more severe damage than WT during DSS treatment, but after DSS withdrawal, *Mbd4^−/−^* and WT mice recover comparably. Together, these results underscore the importance of DNA repair in coping with inflammatory challenges, but also reveal that different DNA repair deficiencies can affect injury and healing processes in different ways.

The primary toxic and mutagenic lesion induced by AOM is *O*^6^MeG that mispairs with thymine. Given that Mbd4 can remove thymine opposite *O*^6^MeG [45], it stands to reason that multiple rounds of Mbd4-mediated futile base excision repair may result in apoptosis. In previous studies we showed that mutant backgrounds with decreased apoptosis immediately after AOM treatment subsequently develop more aberrant crypt foci, and that the number of AOM-induced ACF predicts tumor multiplicity in the AOM/DSS assay [13]. However, here we find no evidence of impaired apoptosis in AOM-treated *Mbd4^−/−^* colon crypts, suggesting that Mbd4 is not a major contributor to futile repair cycles subsequent to AOM damage in the colon. The equivalent levels of ACF in AOM-treated WT and *Mbd4^−/−^* mice further support this finding. One reason why *Mbd4^−/−^* colons may not be more susceptible to AOM-mediated apoptosis is because the colon contains functional *O*^6^MeG DNA methyltransferase (Mgmt), the predominant and efficient repair protein for *O*^6^MeG lesions, thereby eliminating the need for Mbd4 to remove thymine opposite *O*^6^MeG lesions. Nonetheless, this result was somewhat surprising in light of previous studies showing impaired apoptosis following treatment with chemotherapeutic agents in *Mbd4^−/−^* mouse embryonic fibroblasts [45] and small intestinal cells [46]. Several factors could potentially account for the discrepancy of the effect of Mbd4 status on apoptosis observed between our study and previous studies. First, there may be cell or tissue type specific differences in the role of Mbd4 in apoptosis, possibly due to different Mgmt activity levels. There are numerous examples of tissue specific differences in DNA-damage induced apoptosis [66]; one such example is observed in differences in the apoptotic response to DSS treatment in the murine small intestine compared to the colon [67]. Alternatively, the differences could be due to the different genotoxic agents used in these studies. However, Cortellino et al. [45] did observe an impaired apoptosis phenotype with the alkylating agent MNNG which, like AOM, induces *O*^6^MeG lesions. Finally, it is possible that the differences observed may be due to inherent differences in the two independently generated *Mbd4^−/−^* mouse lines used in these studies [43, 45].

It is also possible that Mbd4 may function to suppress AOM/DSS related pathology via a mechanism other than repair of RONS-induced DNA damage. One such possibility involves its physical interaction with the Fas-associated death domain protein (FADD) [47]. FADD is essential for maintaining intestinal homeostasis; mice that are deficient in FADD in the colon epithelium (FADD^IEL-KO^) develop spontaneous colitis [68]. Alternatively, loss of Mbd4-mediated transcriptional repression [48] or active 5MeC demethylation [50] could contribute to the severe phenotypes observed in DSS-treated *Mbd4^−/−^* mice. Although a role for Mbd4 in active demethylation remains controversial [24], a recent study has linked abnormal expression of Mbd4 in breast tumors with aberrant CpG methylation and metastasis [51], suggesting that alternative functions of Mbd4 could in principle influence carcinogenesis.

Given that the increased tumor burden in *Mbd4^−/−^* mice (Figure 2D and 2E) did not appear to arise from a greater induction of ACF by AOM, and that DSS induced considerably more severe inflammation-related colitis symptoms in *Mbd4^−/−^* mice, we addressed the formal possibility that the greater tumor burden observed in *Mbd4^−/−^* mice could be driven by an overactive innate immune response in *Mbd4^−/−^* mice. To test this idea, we repeated the AOM/DSS assay in *Mbd4^−/−^* mice whose hematopoietic cells had been replaced with wild-type hematopoietic cells via adoptive transfer; wild-type mice that had undergone exactly the same procedure were used as controls. Transplantation of *Mbd4^−/−^* mice with WT bone marrow did not rescue the clinical symptoms or reduced survival observed in the AOM/DSS assay. This supports the conclusion that the increased tissue damage observed in DSS-treated *Mbd4^−/−^* mice is not due to an overactive innate immune response by the *Mbd4^−/−^* immune system; instead, it suggests that the *Mbd4^−/−^* colonic epithelium itself is more susceptible to inflammation-induced injury than WT colonic epithelium. Interestingly, a recent study in mice identified two non-synonymous polymorphisms in functional domains of Mbd4 that are associated with significant differences in survival after phosgene-induced acute lung injury, supporting the idea that Mbd4 status of epithelial cells may be important for their response to chemical insult [69].

Despite the recapitulation of the more severe clinical symptoms in transplanted *Mbd4^−/−^* mice in the AOM/DSS assay, transplanted WT and transplanted *Mbd4^−/−^* mice had similar tumor burdens at the time of death (Figure 5C and 5D). If only the difference in tumor burdens between the two genotypes within each experiment is considered, this result would appear to support the conclusion that replacement of *Mbd4^−/−^* hematopoietic cells with WT reduces the tumor burden in AOM/DSS treated *Mbd4^−/−^* mice to WT levels. However, comparing the AOM/DSS assays carried out with the non-transplanted (Figure 2) and transplanted mice (Figure 5), the polyp multiplicity at time of death was not significantly different between transplanted and non-transplanted *Mbd4^−/−^* mice (*p* > 0.74). Instead, the polyp multiplicity at time of death was significantly greater in transplanted WT than non-transplanted WT (*p* < 0.001). Hence, the disappearance of the difference in tumor burden between genotypes in the adoptive transfer AOM/DSS assay is due to an increase in tumor burden in transplanted WT relative to non-transplanted WT animals, rather than a change in the tumor burden in the transplanted versus non-transplanted *Mbd4^−/−^* animals. It is thus not entirely clear whether the lack of difference in tumor burden between genotypes in the transplanted mice is due to a rescue of the cancer prone *Mbd4^−/−^* phenotype, and the similar tumor burden in both genotypes may relate to the radiation exposure used to ablate endogenous BM.

An interesting conclusion to be drawn in comparing the AOM/DSS experiments with transplanted and non-transplanted animals is that in this assay, tumor burden does not necessarily correlate with survival or severity of clinical symptoms. Despite not differing in tumor burden at time of death, the transplanted *Mbd4^−/−^* mice nevertheless had drastically reduced survival time and drastically increased clinical morbidity as measured by weight loss.

In conclusion, the results of this study support and extend our previous findings that loss of DNA repair capacity can dramatically exacerbate tissue injury and carcinogenesis associated with chemically induced colitis. In particular, our finding that Mbd4 deficiency exacerbates the course of experimental colitis suggests that MBD4 status may serve as a useful prognostic indicator for management of ulcerative colitis in human patients.

## MATERIALS AND METHODS

### Mice

All animal experiments were approved by the MIT Committee on Animal Care. Mice were housed in an AALAC-accredited facility on a 12 hr light/dark cycle with food and water provided *ad libitum*. *Mbd4^−/−^* mice (*B6.Cg-Mbd4^tm1Wed/J^*) [43] were purchased from The Jackson Laboratory (Bar Harbor, Maine) at backcross 6 to C57BL/6. *Mbd4^−/−^* mice at backcross 6 (> 98% C57BL/6) were used in the experiment in Figure 2; other experiments used *Mbd4^−/−^* mice at backcross 10 (> 99% C57BL/6). Wild-type C57BL/6 mice from the colony were used as controls. Genotyping was carried out by multiplex PCR with the primers Mbd4-F (GGT AAT GAG ACA TTT GG), Mbd4WT-R (GCA AGT GAA CGT TTT GAT CTG A) and Mbd4Neo-R (CAC GAG ACT AGT GAG ACG TG). The WT and *Mbd4^−/−^* alleles produce 250 and 450 bp products, respectively. Donor mice used for the adoptive transfer assay (*B6.SJL-Ptprc^a^ Pep3^b^/BoyJ*) were purchased from The Jackson Laboratory. These mice are congenic with C57BL/6 and carry a CD45 allele not found in C57BL/6 mice to permit differentiation of donor and recipient cells in adoptive transfer studies by immunostaining.

### AOM/DSS assay

The AOM/DSS assay was carried out as described in [11] (Figure 1A). Colon tumors were initiated via a single intraperitoneal injection of azoxymethane (AOM, 12.5 mg/kg). AOM used in all experiments except the adoptive transfer study was obtained from the Midwest Research Institute NCI Chemical Repository. AOM used in the adoptive transfer study was purchased from Sigma (A5486). Tumor-promoting inflammation was established by episodic dosing of 1.5% dextran sulfate sodium (MP Biomedicals, Solon OH) in drinking water. Pilot experiments were carried out to determine the optimal DSS dose. The same lot of DSS was used for all experiments. Mice were monitored daily and euthanized at the end of study or upon becoming moribund. Euthanasia was carried out by CO_2_ asphyxiation followed by cervical dislocation to ensure humane death. Clinical symptoms were assessed on a six point Disease Activity Index (DAI) scale comprising subscores for stool consistency (0–2), rectal bleeding (0–2) and overall condition (0–2). For each criterion, zero indicates absence of symptoms; 2 indicates severe symptoms. Details of DAI scoring criteria are given in Supplementary Table 1. Mice that succumbed during the first DSS cycle were not included in analysis of polyp multiplicity or area, as these mice would not have had time to develop macroscopic tumors. Image analysis of tumor area was carried out using ImageJ. Two *Mbd4^−/−^* mice that succumbed midway though the experiment depicted in Figure 2 were not included in the image analysis of tumor area because suitable photographs were not available.

### Histopathology

Formalin-fixed tissues were paraffin embedded and H&E stained slides prepared at the MIT David H. Koch Institute for Cancer Research Histology Core Facility. Tissue damage, inflammation, and neoplasia were scored using criteria described in [11] by a board-certified veterinary pathologist (MIT Division of Comparative Medicine) blinded to the identity of the samples.

### Assessment of aberrant crypt foci

Mice were injected intraperitoneally with a single dose of azoxymethane (12.5 mg/kg) and colons were harvested 18 weeks post injection. Colons were stained with 0.5% methylene blue in 70% ethanol and aberrant crypt foci (small raised areas of darker staining) were counted in whole mounts under a stereomicroscope [13].

### Assessment of apoptotic indices

Mice were injected intraperitoneally with a single dose of azoxymethane (12.5 mg/kg) and colons were harvested 48 hours post injection. Apoptosis due to alkylating agents such as AOM that produce *O*^6^MeG lesions occurs on the second cell cycle post exposure [70] and the cell cycle of colonic epithelial cells is approximately 24 hours; therefore, the 48 hour timepoint was chosen. Colons were fixed in 10% neutral buffered formalin and paraffin embedded. Apoptotic cells were identified as pyknotic nuclei in H&E stained sections. A minimum of 50 colon crypts was scored per animal.

### Adoptive transfer

Adoptive transfer of bone marrow hematopoietic cells was carried out based on protocols in [65]. Donor mice (*B6.SJL-Ptprc^a^ Pep3^b^/BoyJ*) were purchased from Jackson Labs (Bar Harbor, Maine). These mice are congenic with C57BL/6 mice but differ at the CD45 locus, allowing donor and recipient cells to be distinguished by immunostaining and flow cytometry. Recipient mice received a split dose of 800 and 400 rads three hours apart in a Gammacell 40 Exactor (Best Theratronics, Ontario, Canada) and immediately received a retro-orbital injection of 2 × 10^5^ donor bone marrow cells. Irradiated mice were maintained in autoclaved caging with autoclaved food and autoclaved water containing 0.025% (v/v) Septra (trimethoprim/sulfamethoxazole suspension). Transplanted mice were monitored three times daily during recovery from the procedure.

### Flow cytometry

Reconstituted mice were evaluated for presence of donor and recipient lymphocytes four weeks after receiving donor bone marrow. Lysed whole mouse blood was stained with 1:50 dilutions of phycoerythrin-conjugated anti-mouse CD45.1 (eBiosciences) to identify donor-derived lymphocytes and APC-conjugated anti-mouse TCRβ (eBiosciences) to identify T cells, which are the most radioresistant lymphocyte subpopulation [65]. Flow cytometry was carried out on a FACScalibur instrument (BD Biosciences) in the MIT David H. Koch Institute for Cancer Research flow cytometry core facility. Data were analyzed using FlowJo software. Percent donor derived cells were determined as the percentage of CD45.1 positive peripheral blood mononuclear cells (PBMCs). Percent donor derived T cells were calculated as the percentage of CD45.1 positive cells that were also TCRβ positive.

### Statistics

Statistics were carried out in GraphPad Prism. Survival curves were compared by log-rank test. Other data were analyzed via Mann-Whitney test. Error bars represent SEM, unless otherwise noted in figure legends.

## SUPPLEMENTARY MATERIALS FIGURES AND TABLES


