# Supplementary material for: The Mbd4 DNA glycosylase protects mice from inflammation-driven colon cancer and tissue injury
Source: Oncotarget. 2016 Apr 13;7(19):28624–36. doi: 10.18632/oncotarget.8721 (PMC5053750; doi:10.18632/oncotarget.8721)
Supplement: Supplementary file 1 [file oncotarget-07-28624-s001.pdf]

The Mbd4 DNA glycosylase protects mice from inflammation-driven colon cancer and tissue injury

Supplementary Materials

Supplementary Table S1: DAI scoring criteria

Diarrhea	0	Stools are normal, small and firm
	0.5	Stools are normal size but sticky.
	1.0	Stools are large and soft, but not liquid.
	1.5	Stools are liquid.
	2.0	Stools are liquid and the mouse's tail/anus is smeared with feces.
Rectal Bleeding	0	No blood observed on rectum or stools.
	0.5	No blood evident on/around rectum, but a small amount on stools.
	1.0	Small amount of blood on rectum, or stools with substantial blood.
	1.5	Substantial amount of blood visible on rectum.
	2.0	Blood on rectum extends to surrounding fur.
Overall Condition	0	Mouse behaves normally and is "bright, alert, reactive"
	0.5	Activity level is normal but mouse may be slightly hunched or waddle.
	1.0	Activity subdued; may have slightly reduced muscle tone; grooming may be reduced, posture may be hunched. Body condition of no less than 2, not dehydrated.
	1.5	Mouse is obviously ill but not moribund. May be dehydrated. Muscle tone may be reduced. Body condition no less than 2−.
	2.0	Mouse is moribund. Extremities are pale or cold; animal is reluctant to move.

Overall DAI is calculated as the sum of scores for all three categories. In all cases, 0 = normal; 2.0 = severe pathology.

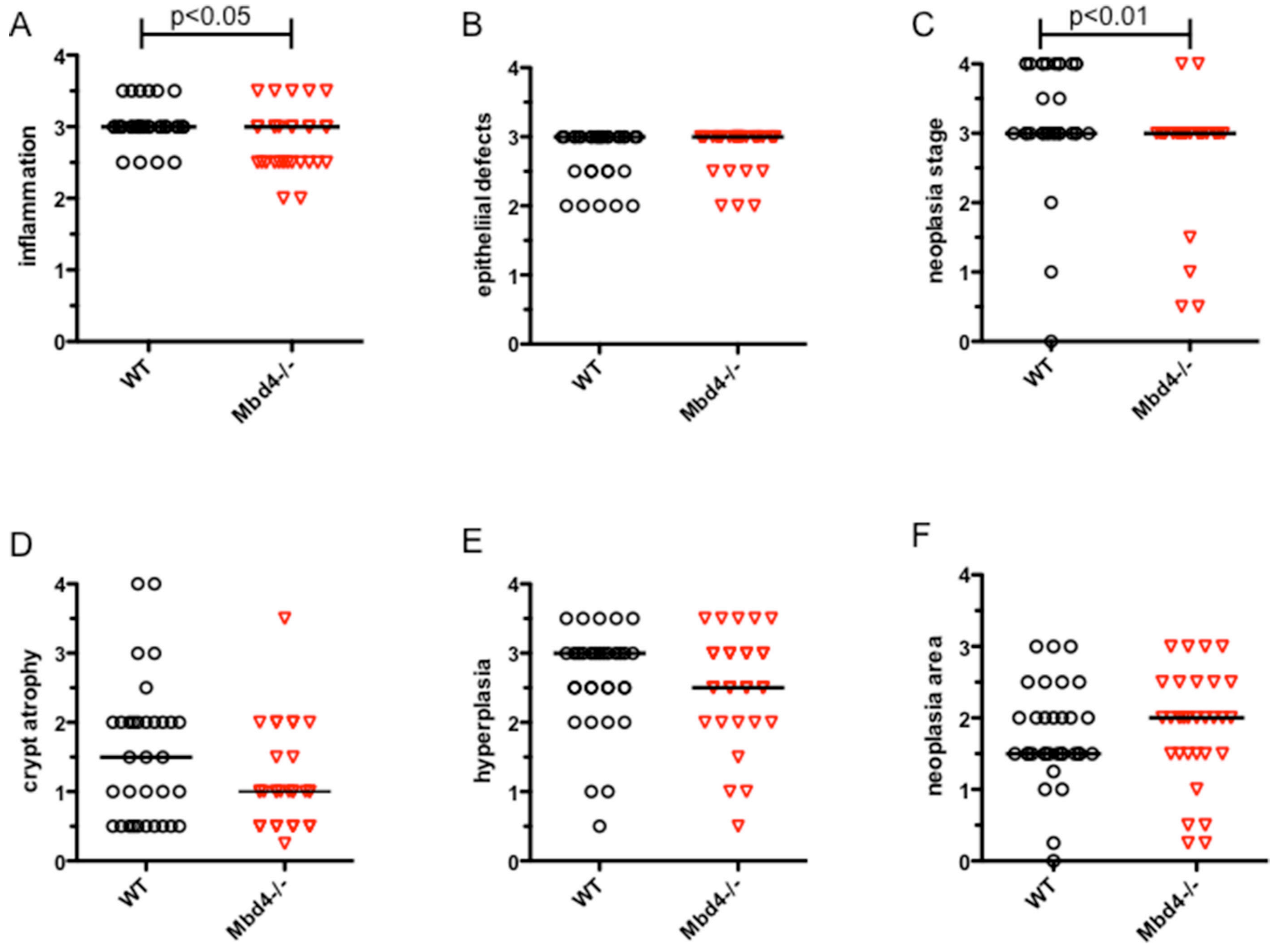

Supplementary Figure S1: Histopathological criteria from tumors in WT (n = 33) and *Mbd4*$^{-/-}$ (n = 30) mice. Mean pathological scores are shown for (**A**) inflammation, (**B**) epithelial defects, (**C**) neoplasia stage, (**D**) crypt atrophy, (**E**) hyperplasia, and (**F**) neoplasia area between genotypes when scored in H&E stained sections.

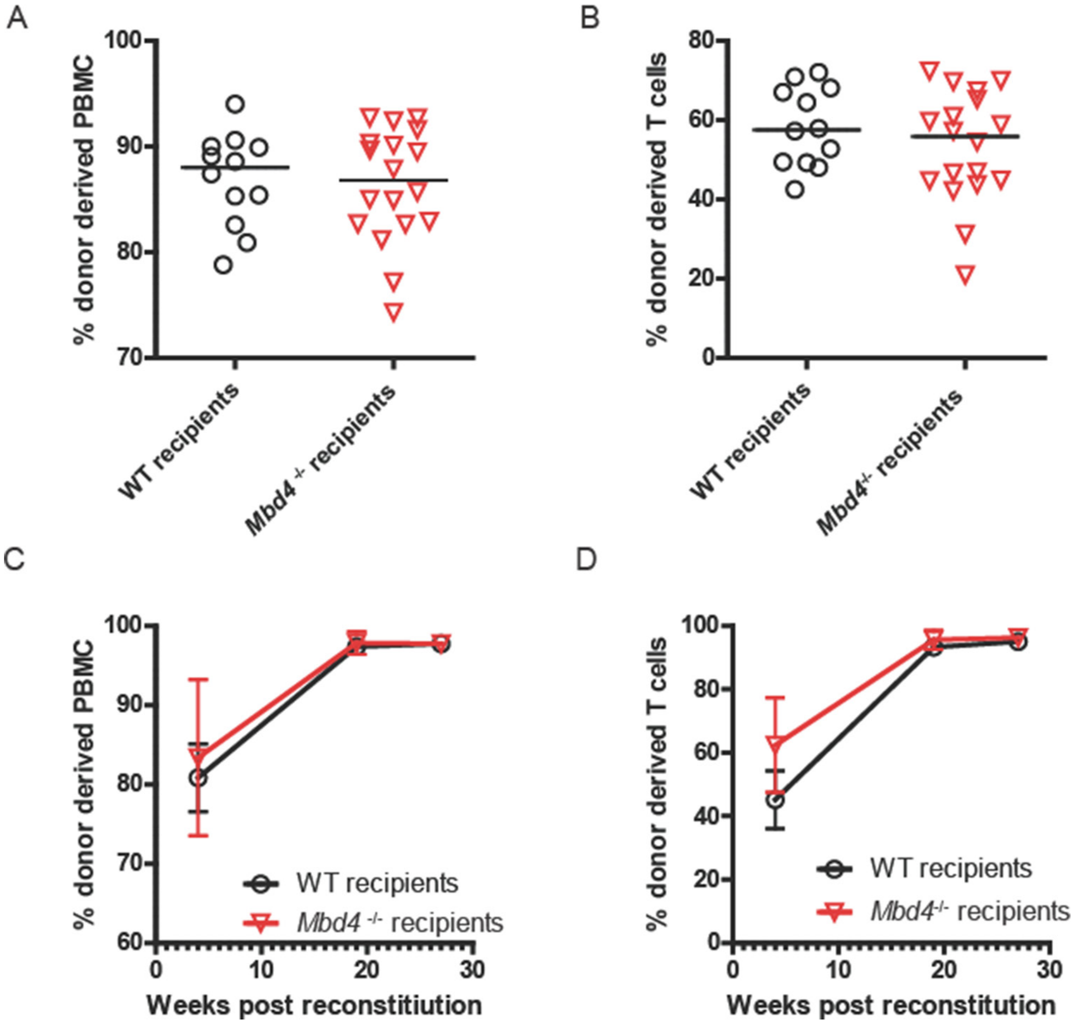

Supplementary Figure S2: Successful adoptive transfer of WT bone marrow into both WT and *Mbd4*$^{-/-}$ mice. (**A**) Flow cytometry analysis of lysed whole blood 4 weeks after the adoptive transfer procedure shows that approximately 75–95% of all peripheral blood monocytes (PBMCs) are derived from donor. (**B**) No difference between genotypes in percent donor-derived T cells 4 weeks after adoptive transfer. Adoptive transfer is stable over time in both WT and *Mbd4*$^{-/-}$ mice. Percent donor-derived total PBMC (**C**) and percent donor-derived T cells (**D**) was assessed at 4, 19, and 27 weeks post reconstitution (n = 3 for both genotypes except that n = 4 for *Mbd4*$^{-/-}$ at the 4 week timepoint).
